# Developing implementation research capacity: longitudinal evaluation of the King’s College London Implementation Science Masterclass, 2014–2019

**DOI:** 10.1186/s43058-020-00066-w

**Published:** 2020-09-11

**Authors:** Rachel Davis, Brian Mittman, Madelene Boyton, Aoife Keohane, Lucy Goulding, Jane Sandall, Graham Thornicroft, Nick Sevdalis

**Affiliations:** 1 Centre for Implementation Science, Health Service and Population Research Department, King’s College London, London, UK; 2 Department of Research and Evaluation, Kaiser Permanente, Pasadena, USA; 3 Department of Women and Children’s Health, School of Life Course Science, King’s College London, London, UK; 4 Centre for Global Mental Health, Health Service and Population Research Department, King’s College London, London, UK

**Keywords:** Implementation science, Capacity building, Training, Course, Evaluation

## Abstract

**Background:**

Despite an increasing number of training opportunities in implementation science becoming available, the demand for training amongst researchers and practitioners is unmet. To address this training shortfall, we developed the King’s College London ‘Implementation Science Masterclass’ (ISM), an innovative 2-day programme (and currently the largest of its kind in Europe), developed and delivered by an international faculty of implementation experts.

**Methods:**

This paper describes the ISM and provides delegates’ quantitative and qualitative evaluations (gathered through a survey at the end of the ISM) and faculty reflections over the period it has been running (2014–2019).

**Results:**

Across the 6-year evaluation, a total of 501 delegates have attended the ISM, with numbers increasing yearly from 40 (in 2014) to 147 (in 2019). Delegates represent a diversity of backgrounds and 29 countries from across the world. The overall response rate for the delegate survey was 64.5% (323/501). Annually, the ISM has been rated ‘highly’ in terms of delegates’ overall impression (92%), clear and relevant learning objectives (90% and 94%, respectively), the course duration (85%), pace (86%) and academic level 87%), and the support provided on the day (92%). Seventy-one percent of delegates reported the ISM would have an impact on how they approached their future work. Qualitative feedback revealed key strengths include the opportunities to meet with an international and diverse pool of experts and individuals working in the field, the interactive nature of the workshops and training sessions, and the breadth of topics and contexts covered.

**Conclusions:**

Yearly, the UK ISM has grown, both in size and in its international reach. Rated consistently favourably by delegates, the ISM helps to tackle current training demands from all those interested in learning and building their skills in implementation science. Evaluation of the ISM will continue to be an annual iterative process, reflective of changes in the evidence base and delegates changing needs as the field evolves.

Contributions to the literature

- Training opportunities in implementation science are increasing, but there is still a shortage of options available to all individuals interested in working in the field.
- We developed the UK Implementation Science Masterclass, the largest course of its kind to address this shortfall. Our findings reflect the success of the ISM and the international growing appetite to learn about implementation science.
- Through our findings, training gaps and priorities were identified together with challenges in developing training in the field. These findings would be useful to consider for those looking to develop future training endeavours in the field.

## Introduction

Implementation science is a rapidly evolving discipline with a significant role in bridging the widely cited ‘research to practice’ gap [1–3]. On average, it takes an estimated 17 years for research findings to be translated into their intended clinical settings [4–6]. Many studies never go beyond publication [7, 8], and of those that do, widespread and systematic implementation of findings is seldom achieved [9, 10]. This consistent failure to efficiently implement evidence into practice not only represents a missed opportunity to improve health outcomes and save lives but also results in significant resource burden for the health and social care system as a whole [10–13]. Uncovering ways to close this quality chasm is fundamental, if health and social care outcomes are to be improved [9, 14, 15].

The field of implementation science has experienced significant growth over the last two decades [1, 16, 17]. As interest in the field has increased [1], so has the appetite of researchers and implementers (those tasked with implementing healthcare evidence) to learn about implementation science methodologies [18–22]. To keep abreast with these demands, opportunities for building capacity in implementation science are essential [19, 20, 23–25]. The field has responded to this increased demand—such that a variety of teaching initiatives and training programmes have emerged [3, 17, 21, 26–28]. Typically, these take the form of webinars or short courses and ‘taster’ sessions over 1–5 days [29–33] but may also form part of masters, doctoral, or post-doctoral programmes [21, 34–37]. The USA and Canada have paved the way in many of these efforts with the establishment of training institutes [25–28, 38], academic courses and certificate programmes [10, 35, 39–44], and webinar series [45, 46]. Training opportunities in the UK [47, 48], other parts of Europe [21, 37], Asia [49], Australia [29, 50], and Africa [51] have also been reported, along with the recent development of a Massive Open Online Course (MOOC) in Implementation Research by the Special Programme for Research and Training in Tropical Diseases (TDR) [52].

In 2015, *Implementation Science* highlighted a renewed interest in manuscripts describing and appraising education and training offerings [23] (such as those described), so that the educational effectiveness of the training courses and events on offer can be formally appraised. Further to this, in 2017, a review of dissemination and implementation capacity-building initiatives in the USA raised the importance of formal evaluation to ensure users’ needs are being met and to inform the planning of future initiatives [18].

Research in the field on the impact of training on knowledge acquisition, understanding, and interest has shown considerable promise. Taken collectively, findings demonstrate the value in preparing new researchers to conduct implementation research, upskilling those already working in the field, increasing confidence in the application of acquired skills, and forging working relationships between multidisciplinary audiences [2, 17, 26–28, 33, 51, 53]. While findings are positive, much of the evidence to date is USA- and Canada-centric [3, 26–28, 31–34, 38, 53, 54], with fewer evaluations of training endeavours in other parts of the world [21, 29, 37, 51] and a noticeable gap in evidence within the UK context.

This paper aims to build on the existing literature and address this evidence gap. We report an innovative UK training initiative, which to the best of our knowledge is the largest of its kind in Europe: the ‘Implementation Science Masterclass’ (ISM), led by the Centre for Implementation Science, King’s College London. We describe the course development and delivery and report on delegates’ evaluations and faculty reflections over its first 6 years (2014–2019).

## Methods

### Institutional context

The ISM is partly funded by the National Institute of Health Research’s (NIHR) Applied Research Collaboration (ARC) South London, 2019–2024 (formerly the ‘Collaboration for Applied Leadership Health Research and Care’ (CLAHRC) South London, 2013–2018). This is one of a number of applied health research collaborations across England, centrally funded by the NIHR to produce highly implementable and high-quality applied health research that addresses urgent health and healthcare needs of the English population, and also to develop capacity in applied health research and healthcare implementation (including implementation research and practice). The NIHR ARC South London comprises a diverse and multidisciplinary team working collaboratively between academic institutions and NHS organisations across South London.

Within this research infrastructure, the Centre for Implementation Science was established in 2014 and became operational in 2015. Based within the Health Service and Population Research Department of King’s College London, the Centre leads implementation research and education activities and supports the research carried out by the ARC South London with the primary goal of helping to better implement research-driven best practices. The Centre is currently the largest implementation research infrastructure of its kind in the UK and Europe, hosting over 50 staff members—including faculty, scientists, and managerial and administrative personnel. The Centre is the organisation that hosts and delivers the ISM.

### Rationale and aims of the ISM

The ISM was firstly conceptualised in 2013, as part of a drive to augment the UK capacity and capability in implementation research and practice. At the time of inception (2013), there was a significant shortage of UK training opportunities for those interested in learning how to better implement research findings into clinical practice [55]. Training opportunities offered outside the UK (past and present) have not been able to address the training need—many are/have been restricted to small numbers (e.g. capped at 12–20 individuals [26, 27, 38, 56]), involve a competitive application process [26, 27, 36, 38, 56], or are restricted to specific professions [23, 36], contexts [27, 29, 38], specific stage of career [27, 28, 35, 38, 49, 54], or those with established opportunities to conduct an implementation research project in their workplace [10, 36].

The ISM’s goal was to overcome these barriers and address the implementation research capacity shortage in the UK—and potentially also Europe. The primary aim of the ISM was to provide a training mechanism for all individuals interested in the application of implementation science methodologies and techniques, irrespective of their professional background, where they fall on the career trajectory, or their expertise. Secondary to this, we wanted to help encourage collaborative work through developing a network of implementation scientists from diverse disciplines, professions, work settings, and socio-demographics.

### ISM annual development and delivery cycle

The ISM is delivered annually, in July, in London, over 2 full days. All aspects of the ISM are delivered face-face, excluding situations where there are unanticipated issues with a speaker’s ability to attend in person; in these circumstances, video conferencing is used. The development and delivery of the ISM follows an annual multiphase, iterative educational cycle within the UK academic year:

*Development stage (September to January)*: the ISM core content and faculty composition for the upcoming July delivery are reviewed and agreed in light of the preceding year’s evaluation and faculty reflections. In the ISM’s first development iteration (2013–2014), this stage also included a needs assessment of healthcare professionals and a curriculum mapping exercise of other relevant training initiatives in order to establish research and education priorities. This activity is now undertaken periodically to ensure the ISM remains relevant and addresses current needs in the UK (and further afield).

*Delivery stage (January to July)*: the ISM is fully planned—including detailed description of the learning objectives of the core elements of the course; description of the course specialist elements; specification of interactive workshops; faculty formulation; finalisation of length, structure, and pedagogical approach(es); and administrative and communications arrangements for the course (incl. course location, communications materials, and handouts). Early in the calendar year, the course registration also becomes available for delegates.

*Evaluation phase (July to August)*: this relates to the collection and analysis of (1) delegates’ evaluations of the ISM (July), along with (2) faculty reflections (July to August), which are then used to inform the content and structure of the course for the following year—hence closing an iterative annual feedback and learning loop.

*Delegates’ evaluations:* a structured and standardised evaluation form is used to assess delegates’ overall impression of the ISM as well as attitudes towards the ISM’s relevance and clarity of learning objectives, the appropriateness of its pace, duration and academic level, and the level of support provided by the faculty and organising committee before the ISM and its 2-day duration. Evaluation forms are included in the information pack delegates receive on the first day of the ISM. Delegates are encouraged to complete and hand in the evaluation survey at the end of the ISM: for those unable to do this, the survey is circulated via email, the day after the ISM. Responses are provided on a 5-point ordinal scale (e.g. ‘What was your overall impression of the course?’—range—‘very poor’, ‘poor’, ‘fair’, ‘good’, ‘very good’) or 3-point ordinal scale (e.g. ‘What did you think of the course duration?’—‘too short’, ‘about right’, and ‘too long’). For the purpose of the evaluation, the positive and negative anchors derived from the 5-point scales were amalgamated (e.g. ‘very good’ and ‘good’ were combined to make ‘good’).

Delegates are also given the opportunity to provide free-text feedback on perceived key strengths of the ISM and what they felt could be done differently. Data is aggregated and fully anonymized for yearly ISM evaluation reports for the Centre for Implementation Science. Individual data are then destroyed due to General Data Protection Regulations (GDPR), a set of data protection rules implemented in UK law as the Data Protection Act 2018 (https://www.legislation.gov.uk/ukpga/2018/12/contents/enacted).

*Faculty reflections:* the ISM faculty are annually invited to take part in two debriefings: one ‘hot debrief’ and one ‘cold debrief’. The hot debrief takes place upon completion of the ISM—in a faculty group session or in smaller groups, facilitated by one of the two ISM co-directors. The aim is to capture initial thoughts and the experience of the course immediately upon its completion that will otherwise be forgotten or be filtered through subsequent reflection. The cold debrief is always virtual. The faculty are invited to submit their reflections on the course in August and September through a faculty group email, facilitated by one of the two co-directors. All emails are collected and collated, and then submitted to the ISM organising committee at their first meeting for the subsequent year’s ISM (typically in September to October annually), alongside the detailed evaluation report, which includes a summary of the delegates’ ISM evaluations. Annual ISM improvements are driven by these faculty reflections and the delegates’ summary evaluation report.

### Faculty and organising committee

The faculty represent a range of countries and continents, professional backgrounds (e.g. medics, researchers, service users, and patient representatives), disciplines (e.g. psychology, psychiatry, public health, medicine, and epidemiology), work settings (e.g. academic or healthcare organisations and services), and health services (e.g. cancer, surgery, diabetes). Taken collectively, the faculty hold expertise spanning all areas of implementation science.

The organising committee is chaired by 2 academic co-directors and includes experienced implementation scientists, communication officers, education programme managers, and experienced administrators. The committee oversees the scientific direction of the ISM, procurement and allocation of resources, substantive iterations to content (year on year), and dissemination of relevant information, materials, and guidance to the wider faculty and delegates (see Table 1 for a full list of the faculty over the 6 years of the ISM).

**Table 1:** ISM faculty 2014–2019

Name	Title	Affiliation	Years involved
Greg Aarons	Professor of psychiatry	University of California, USA	All 6
Ricardo Araya	Professor of global mental health	King’s College London, UK	6
Sarah Birken	Assistant professor in the Department of Health Policy and Management	Gillings School of Global Public Health, USA	6
Annette Boaz	Professor of healthcare research	Kingston University and St George’s, University of London, UK	4, 5, 6
Geoff Curran	Professor of pharmacy practice and psychiatry	University of Arkansas for Medical Sciences, USA	5, 6
Richard Emsley	Professor of medical statistics and trials methodology	King’s College London, UK	6
Jill Francis	Professor of health services research	City, University of London, UK	4
Kim Goldsmith	Biostatistician and clinical trials specialist	King’s College London, UK	6
Lucy Goulding*	Kings improvement science programme manager	King’s College London, UK	2, 3, 4, 5
Bridie Kent	Professor in leadership in nursing	Plymouth University, UK	1
Peter Littlejohns	Professor of public health	King’s College London, UK	5, 6
Susan Michie	Professor of health psychology	University College London, UK	3
Brian Mittman*	Senior research scientist	Kaiser Permanente, USA	All 6
Joanna Moullin	Lecturer in the faculty of health sciences	Curtin University, Australia	6
Per Nilsen	Professor of social medicine and public health	Linköping University, Sweden	5, 6
John Ovretveit	Professor of healthcare innovation	Karolinska Institute, Sweden	2, 4, 5, 6
Anne Rogers	Professor of health systems implementation	University of Southampton, UK	3, 4, 5, 6
Diana Rose	Professor of user-led research	King’s College London, UK	2
Anne Sales	Professor in the School of Medicine	University of Michigan, USA	1, 3
Jane Sandall	Professor of women’s health	King’s College London, UK	3, 4, 5, 6
Nick Sevdalis*	Professor of patient safety and implementation science	King’s College London, UK	2, 3, 4, 5, 6
Rahul Shidhaye	Clinical psychiatrist	Ruby Hall Clinic, India	2
Sharon Strauss	Professor in the Department of Medicine	University of Toronto, Canada	1, 2, 3
Graham Thornicroft*	Professor of community psychiatry	King’s College London, UK	1, 2, 3

*Individuals that have been part of the organising committee

### Course development, curriculum, and structure

Five guiding principles underpin the ISM curriculum: (1) to have relevance to both researchers and implementers; (2) to focus on different health and care contexts, locally, nationally, and internationally, to illustrate that implementation issues are endemic in any care setting; (3) to take a ‘systems’ approach to highlight that implementation is a multi-level phenomenon (e.g. involving individuals, teams, organisations); (4) to comprise a mixture of teaching approaches and interactive sessions to meet the educational needs of new and intermediary learners as well as those with more expertise; and (5) to enable formal and informal interactions with faculty and delegates to forge experiential and transdisciplinary understanding of the methodological issues and conceptual challenges within the field.

Based on these principles, the ISM curriculum follows a 4-block structure, with each block delivered within a half-day session. The blocks cover the following broad thematic areas:

- *Block 1—introduction to implementation science*: delivered as the first half-day of the ISM; this block introduces the delegates to the basic concepts and definitions of the science, its historic provenance, and how it differs from/relates to other areas of health research (e.g. clinical effectiveness research).
- *Block 2—implementation theories and frameworks*: this block exposes delegates to theoretical frameworks developed and used by implementation scientists, including exemplar applications within research studies.
- *Block 3—implementation research and evaluation methods and designs*: this block covers research design elements applicable to implementation research, including an introduction to hybrid effectiveness-implementation designs, other trial and observational designs of relevance to implementation research questions, the Medical Research Council Framework for evaluation of complex health interventions, process evaluation approaches, and the use of logic models/theory-of-change methodology in implementation studies.
- *Block 4—specialist topics*: the final half-day of the ISM varies on an annual basis, thus offering the opportunity to cover prominent or topical implementation research issues. To date, this block has featured (amongst other topics) sessions on how implementation science relates to improvement science and knowledge mobilisation, the advantages and disadvantages of different implementation research designs, and the usefulness of implementation research for practical policy-making applications. The format of this block is accordingly flexible.

To deliver the four ‘blocks’, the ISM comprises a mixture of plenary lectures, workshops and breakout sessions, and debate panels. The plenaries and lectures describe and discuss the conceptual foundations and methodologies of implementation science. The workshops and breakout sessions focus on the application of specific tools and techniques. The debate panels address current controversies or ‘hot topics’ in implementation science as well as the relationship between implementation science and the related field of applied health research.

The content of the ISM was initially developed by drawing on the core faculty’s extensive research and education expertise. In addition, we reviewed published resources including a list of established core competencies in knowledge translation [25], taken from a 2011 Canadian training initiative, as well as a 2012 framework for training healthcare professionals in implementation and dissemination science [41]. Newer evidence in the field (i.e. post-inception of the ISM in 2013) is considered yearly and used to help inform the following year’s curriculum as needed: examples include published evaluations of implementation science training initiatives [21, 27, 28, 31, 32, 34, 37, 38, 54], the National Implementation Research Network’s recent 2018 working draft of core competencies for an implementation practitioner [57], and a scoping review of core knowledge translation competencies [58]. The ISM involves no summative assessment, but understanding is assessed formatively through the interactive group-based sessions.

Registration for the ISM has been through a simple online process with places offered on a first-come-first-served basis.

Further information on any aspect of the ISM is available from the lead author (RD) upon request.

## Results

Following the initial course in July 2014, the ISM is held annually in London, UK. The course is currently in its sixth year of running, with the 2020 ISM fully scheduled.

Each year, the number allowed to register has increased, to account for growing demand: starting from 40 delegates in 2014, with the most recent year (2019) capped at 150. A standardised fee structure is applied, in tandem with the host academic institution’s (KCL) short course fee structure to ensure transparency and equity. Discounted fees are offered to specific groups of individuals (e.g. low- and middle-income country (LMIC) nationals, service users, those working in non-governmental organisations (NGOs) and/or within the ARC South London footprint).

### Information on delegates

To date, 501 delegates from over 29 countries have attended the masterclass. Most delegates (75%) have been UK-based (380/501) with 51% (258/501) residing in London, UK. The number of overseas delegates has increased year-on-year, from 13% (*N* = 5/40) in year 1 (2014) to 33% (*N* = 48/147) in year 6 (2019) (see Table 2).

**Table 2:** Delegate information

Year	Delegates, *N*	London-based, *N* (%)	Other parts of the UK, *N* (%)	Overseas, *N* (%)	Countries of overseas delegates, *N* (%)
2014*	40	29 (73)	6 (15)	5 (13)	India, Spain, USA, Canada
2015*	46	32 (70)	7 (15)	7 (15)	Ireland, Denmark, Switzerland, Canada, Japan, India
2016	80	45 (56)	23 (29)	12 (15)	Denmark (3), Netherlands (2), Ireland (2), Germany (1), Belgium (1), Jamaica (1), Switzerland (1)
2017	87	46 (53)	24 (28)	17 (20)	Norway (5), Germany (3), Denmark (2), Portugal (2), Ireland (1), Italy (1), New Zealand (1), Malaysia (1), South Africa (1)
2018	101	41 (41)	28 (28)	32 (32)	Australia (6), Netherlands (5), USA (4), Ireland (4), Norway (3), Czech Republic (2), Belgium (1), Canada (1), France (1), Germany (1), New Zealand (1), South Africa (1), Sweden (1), Switzerland (1)
2019	147	65 (44)	34 (23)	48 (33)	Australia (11), Denmark (1), Germany (4), Hong Kong (1), Ireland (3), Italy (1), Jordan (1), Malta (1), Netherlands (6), New Zealand (2), Nigeria (1), Norway (3), Russia (1), South Africa (1), Sweden (2), Switzerland (4), Taiwan (1), Uganda (1), USA (3)
Total	501	258 (51)	122 (24)	121 (24)	

*We do not have the individual breakdown of data for 2014/2015. Data was taken from the aggregated and fully anonymised annual evaluation of the ISM report. Individual-level data was destroyed after the reports had been written

There were a few dropouts each year due to extraneous circumstances, e.g. the 2019 ISM had 150 registered, 147 attended (dropouts due to illness, etc.)

The course attracts delegates from a range of cultural, ethnic, and professional backgrounds (e.g. nurses, doctors, healthcare managers, psychologists, economists, policy makers, patient representatives, and epidemiologists), and different stages of their career (e.g. doctoral students, post-doctoral fellows, research staff, junior, and senior faculty), representing multiple academic departments, including social work, public health, medicine, pharmacy, and psychology, as well as those from non-academic health and social care organisations.

### Delegates’ evaluations of the ISM

The overall response rate from delegates across the six years was 64.5% (323/501). Table 3 displays the breakdown of data in relation to the evaluation survey. An overwhelming majority of delegates (92%, 294/318 responses) reported that their overall impression of the course was ‘good’ or ‘very good’. The majority also stated that the learning objectives were relevant (94%, 287/306), clear (90%, 277/307), and reached 84% (256/306). Seventy-four percent of delegates (233/313) rated the pre-course reading material favourably, with most agreeing that the pace of the course (86%, 275/320), the course duration (85%, 271/319), and the academic level (87%, 275/316) were ‘about right’*.* Seventy-one percent (219/307) felt that the ISM would have an impact (‘definitely’ or ‘partly’) on how they approach their practice and/or future research, with the majority also rating both the level of pre-course support for delegates and support during the day as ‘high’ (89%, 276/310, and 92%, 281/304, respectively).

**Table 3:** Delegates’ evaluations of the masterclass

	2014, *N* (%)	2015, *N* (%)	2016, *N* (%)	2017, *N* (%)	2018, *N* (%)	2019, *N* (%)	Total, *N* (%)
Overall impression	Good: 20 (95) Fair: 1 (5) Poor: 0 (0)	Good: 32 (97) Fair: 1 (3) Poor: 0 (0)	Good: 36 (97) Fair: 1 (3) Poor: 0 (0)	Good: 51 (96) Fair: 2 (4) Poor: 0 (0)	Good: 75 (91) Fair: 7 (9) Poor: 0 (0)	Good: 80 (87%) Fair: 9 (10%) Poor: 3 (3)	Good: 294 (92) Fair: 21 (7) Poor: 3 (1)
Learning objectives clear	Yes: 20 (95) Partly: 1 (5) No: 0 (0)	Yes: 32 (100) Partly: 0 (0) No: 0 (0)	Yes: 37 (95) Partly: 2 (5) No: 0 (0)	Yes: 48 (94) Partly: 3 (6) No: 0 (0)	Yes: 78 (95) Partly: 3 (4) No: 1 (1)	Yes: 62 (76) Partly: 15 (18) No: 5 (6)	Yes: 277 (90) Partly: 24 (8) No: 6 (2)
Learning objectives relevant	Yes: 19 (90) Partly: 2 (10) No: 0 (0)	Yes: 32 (100) Partly: 0 (0) No: 0 (0)	Yes: 40 (100) Partly: 0 (0) No: 0 (0)	Yes: 49 (98) Partly: 1 (2) No: 0 (0)	Yes: 78 (98) Partly: 2 (3) No: 0 (0)	Yes: 69 (83) Partly: 12 (14) No: 2 (2)	Yes: 287 (94) Partly: 17 (5) No: 2 (1)
Learning objectives reached	Yes: 19 (90) Partly: 2 (10) No: 0 (0)	Yes: 31 (97) Partly: 1 (3) No: 0 (0)	Yes: 33 (85) Partly: 6 (15) No: 0 (0)	Yes: 43 (86) Partly: 6 (12) No: 1 (2)	Yes: 71 (89) Partly: 8 (10) No: 1(1)	Yes: 59 (70) Partly: 21 (25) No: 5 (5)	Yes: 256 (84) Partly: 44 (14) No: 6 (2)
Pre-course reading material	About right: 19 (90) Too light: 0 (0) Too much: 2 (10)	About right: 29 (91) Too light: 2 (6) Too much: 1 (3)	About right: 32 (82) Too light: 0 (0) Too much: 7 (18)	About right: 33 (66) Too light: 0 (0) Too much: 17 (34)	About right: 64 (78) Too light: 1 (1) Too much: 17 (21)	About right: 56 (63) Too light: 0 (0) Too much: 33 (37)	About right: 233 (74) Too light: 3 (1) Too much: 77 (25)
Pace of the course	About right: 17 (81) Too slow: 1 (5) Too fast: 3 (14)	About right: 28 (88) Too slow: 0 (0) Too fast: 4 (13)	About right: 33 (83) Too slow: 0 (0) Too fast: 7 (18)	About right: 42 (79) Too slow: 1 (2) Too fast: 10 (19)	About right: 76 (93) Too slow: 4 (5) Too fast: 2 (2)	About right: 79 (86) Too slow: 2 (2) Too fast: 11 (12)	About right: 275 (86) Too slow: 8 (2) Too fast: 37 (12)
Course duration	About right: 14 (67) Too long: 6 (29) Too short: 1 (5)	About right: 28 (88) Too long: 4 (13) Too short: 0 (0)	About right: 37 (93) Too long: 2 (5) Too short: 1 (3)	About right: 42 (81) Too long: 7 (13) Too short: 3 (6)	About right: 73 (89) Too long: 3 (4) Too short: 7 (6)	About right: 77 (85) Too long: 4 (4) Too short: 10 (11)	About right: 271 (85) Too long: 26 (8) Too short: 22 (7)
Academic level	About right: 19 (90) Too low: 0 (0) Too high: 2 (10)	About right: 32 (100) Too low: 0 (0) Too high: 0 (0)	About right: 36 (90) Too low: 0 (0) Too high: 4 (10)	About right: 42 (81) Too low: 2 (4) Too high: 8 (15)	About right: 67 (82) Too low: 6 (7) Too high: 9 (11)	About right: 79 (89) Too low: 2 (2) Too high: 8 (9)	About right: 275 (87) Too low: 10 (3) Too high: 31 (10)
Impact of course on work	Yes: 16 (76) Partly: 5 (24) No: 0 (0)	Yes: 27 (87) Partly: 3 (10) No: 1 (3)	Yes: 26 (68) Partly: 11 (29) No: 1 (3)	Yes: 36 (68) Partly: 17 (32) No: 0 (0)	Yes: 56 (72) Partly: 21 (27) No: 1 (1)	Yes: 58 (65) Partly: 29 (33) No: 2 (2)	Yes: 219 (71) Partly: 86 (28) No: 5 (2)
Pre-course support	Good: 18 (90) Fair: 2 (10) Poor: 0 (0)	Good: 28 (91) Fair: 3 (10) Poor: 0 (0)	Good: 36 (94) Fair: 2 (5) Poor: 0 (0)	Good: 48 (91) Fair: 4 (8) Poor: 1 (2)	Good: 70 (87) Fair: 8 (10) Poor: 2 (3)	Good: 76 (86) Fair: 8 (9) Poor: 4 (5)	Good: 276 (89) Fair: 27 (9) Poor: 7 (2)
Support during the day	Good: 20 (95) Fair: 1 (5) Poor: 0 (0)	Good: 29 (94) Fair: 2 (6) Poor: 0 (0)	Good: 31 (97) Fair: 1 (3) Poor: 0 (0)	Good: 49 (93) Fair: 2 (4) Poor:2 (4)	Good: 77 (97) Fair: 3 (4) Poor: 0 (0)	Good: 75 (86) Fair: 11 (13) Poor: 1 (1)	Good: 281 (92) Fair: 20 (7) Poor: 3 (1)
Overall response rate (%)*	21/40 (53%)	34/46 (74)	40/80 (50)	53/87 (61)	82/101 (80)	93/147 (63)	323/501 (64.5)
RR range	20–21/40	31–33/46	32–40/80	50–53/87	78–82/102	82–93/147	304–320/501

Percentages are rounded down if < .5

*Overall response rate calculated using the number of delegates that completed the evaluation survey, irrespective of missing data (i.e. if they did not complete all questions)

Free-text feedback on the ISM was provided by more than half (> 65%) the delegates. Comments on the ‘strengths’ of the course across all 6 years focused on the following:

- *Breadth and variety of topics*: e.g. ‘nice breadth of topics’, ‘comprehensive overview of the field’.
- *Quality of the speakers*: e.g. ‘the speakers were all excellent. Very engaged and helpful’, ‘clearly incredibly knowledgeable and approachable’.

*Benefit of interactive sessions*: e.g. ‘it was useful to apply the learnings from the lectures in the workshops’, ‘very valuable having the small group sessions’, ‘the discussions gave me the chance to really understand and apply the content being presented’.

*Networking opportunities*: e.g. ‘interacting with experts in the field, meeting colleagues and networking informally’, ‘I loved having the opportunity to interact with researchers in the field and editors of journals’.

*Diversity of faculty and audience*: e.g. ‘speakers from a variety of fields’, ‘getting the perspectives of researchers internationally’, ‘global perspective of implementation science from a diversity of fields’.

*Consolidation of learning*: e.g. ‘enough repetition and continuity to embed ideas’, ‘reinforced concepts I was familiar with and stretched my thinking about challenges and questions to be answered’, ‘validated what I understood already’.

*Structure*: e.g. ‘mixture of presentations and group-based sessions’, ‘combination of lectures followed by workshops’.

Comments on how the ISM could be improved in the future centred on the following:

*Greater focus and separation on LMIC and other contexts*: e.g. ‘I don’t think I was the right person for the course because I work in LMIC and did not feel the content was relevant to these contexts’, ‘the focus on healthcare – I would have liked to have seen more relevance to social care’, ‘it would have been useful to separate out local and global streams’.

- *Opportunities to discuss own work*: e.g. ‘more time for small group work to discuss individual projects’, ‘some project clinic time for reflection on specific projects’, ‘more opportunities to talk about my own work’, ‘clinic to discuss own projects with experts’.
- *Greater interactivity*: e.g. ‘would have liked it to be more interactive’, ‘even more group-based sessions’, ‘greater opportunities to ask questions and engage with tasks’.
- *Support required in the application of knowledge*: e.g. ‘I have learned a lot but I am not able to implement the knowledge’, ‘I know what implementation science is now but am still unsure how to apply it to my field’.

### Faculty reflections

The faculty reflections have focused on several areas over the years—summarised as follows:

*Educational delivery methods*: balance of didactic lectures and workshops; this is a recurring theme. The ISM has always included a mixture of didactic and interactive components. The balance of them as well as the nature of the activities carried out during the interactive workshops has been regularly reflected upon. The faculty have remained keen on the mixture of activities to continue. The interactive workshops have changed in nature—from sessions where the contents of the immediately preceding lecture were reflected upon and discussed in the context of specific delegates’ projects, to delegates submitting summary projects which were themed and reviewed at the workshops, to, more recently, the workshops focusing on specialist topic areas that require a hands-on interactive approach—including how to publish implementation studies, how to apply the concept of a learning healthcare system, and how to carry out stakeholder engagement activity and other specialist topics.

*ISM educational level*: introductory vs advanced curriculum streams; in its early years and following the original needs assessment, the ISM was designed as a plenary course—i.e. all delegates were always kept together in plenary sessions, and attended similarly themed workshops. However, over the years, this approach shifted—such that as of 2019 the ISM includes two streams, one aimed at introductory learners and the other aimed at advanced learners. The lectures and workshops are themed accordingly for these two streams.

*Selection and prioritisation of topic areas within the curriculum*: the faculty have been very keen on keeping the curriculum relevant but also refreshing it annually. This has meant that the curriculum has gravitated towards coverage of key methodological aspects of implementation science (including hybrid designs, implementation theories and frameworks, application of theory of change/logic model methodology, and complex intervention evaluation design). At the same time, the need to refresh the ISM and keep it current has meant that a number of specialist areas have also been covered over the years—including the interface of implementation with improvement science and knowledge mobilisation, funding implementation research, and implementation research in the context of global health.

*Provision of project (incl. papers, grants, service implementation projects) development support and mentoring*: the faculty have often reflected that some of the more advanced learners who are at a stage of their careers at which they are submitting funding bids or designing implementation studies would benefit from a mentoring scheme to be delivered through the ISM. A pilot operationalization of this reflection will be offered as part of the 2020 ISM.

### Examples of iterations to the course

The process of designing and delivering the ISM each year is a continual process. Key changes we have made centre on the registration process, the setting, the content, and the structure. For example, after the 2015 ISM, we streamlined the registration process so that delegates no longer had to submit an abstract (which detailed an outline of an implementation project for discussion and development) because acted as a deterrent to some, especially if they were new to the field. After the 2014 ISM, we changed the venue and room layout after many individuals commented on how the workshops and group work felt fragmented and broken up by walking to different venues. In 2016, after delegates stated that they were ‘hoping for more on evaluations’, we incorporated sessions on evaluating complex interventions in the curriculum for the following years. We have, over the years, also reduced the number of didactic classes per day and increased (where possible) formal and informal opportunities to network and discuss research projects with faculty and peers. In 2019, we also introduced an advanced implementation science stream (mentioned in the faculty reflections above) based on requests from a growing number of delegates and the need to cater for their differing levels of expertise.

### Wider impact and evolution of additional training offerings

We have developed several other training initiatives that have stemmed either directly or partly from feedback on the ISM. Key outputs include the following:

*Project advice clinics*—we offer bespoke advice clinics, introduced (in 2017) as a direct result of repeated and growing numbers of requests from ISM delegates for feedback on their own implementation science projects. Anyone can apply, and clinics run all through the year, providing real-time feedback to individuals as and when they need it. To date, we have conducted 39 clinics (2017 = 15, 2018 = 13, 2019 = 11); though these figures are not an indicator of the demand for the clinics, rather they are reflective of the capacity we have had to deliver them. Feedback collected from attendees has been extremely positive in terms of the usefulness of the clinics and level of support provided.

*UK Implementation Science Annual Research Conference*—as the ISM has grown, it has become harder to accommodate delegates’ desires to showcase and discuss their own research in the group-based sessions. As a direct expressed need, we developed the UK Implementation Science Annual Research Conference, which is the largest conference of its kind in the UK (and will be in its 3rd year in 2020). In 2018, 116 delegates attended the conference from 16 countries; this number increased to 148 in 2019, with delegates from 17 countries, including (but not limited to) Austria, Belgium, South Africa, Spain, New Zealand, USA, Canada, Sweden, Nigeria, Taiwan, and Hong Kong.

- *Additional offerings*—we have developed a range of lectures and half or full day training offerings embedded within formal postgraduate courses at King’s College London (e.g. the Masters in Public Health and MSc Global Mental Health). These offerings were driven, in part, by feedback from ISM delegates regarding the lack of training opportunities in implementation science. We have also designed and delivered a range of bespoke courses for health and social care organisations. Typically (but not always), these requests originate from individuals that attend the ISM that wish to offer similar training to their host organisation, so we then deliver training tailored to their specific needs and context.

## Discussion

This paper describes the development and evaluation of the UK ISM, led annually by the Centre for Implementation Science, King’s College London in London, UK. Over the 6-year period, the ISM has grown considerably, both in size and in terms of its international reach with delegates attending from all over the world. Across the evaluation, consistently favourable results were reported in terms of knowledge gained, relevance of content, and potential impact on future work. Noteworthy strengths included the breadth of the curriculum and opportunities to network with individuals from a diversity of backgrounds. Several areas of improvement were identified, including allowing more time for group discussions and placing greater emphasis on implementation science in low- and middle-income countries (LMICs) and social care contexts.

The ISM was developed to address the significant lack in training opportunities in implementation science in the UK. At its inception (and currently still), it is the largest initiative of its kind in the UK that provides training for all individuals irrespective of their professional background, qualifications, and expertise or where they fall on the career trajectory. The substantial breadth of topics covered together with the cross-disciplinary, international composition of faculty and delegates provides a rich and varied training environment as well as helping to foster collaborative opportunities.

Our findings, consistent with analogous research [26–28, 33, 37, 38, 54], demonstrate the need and value of training initiatives in implementation science. A key strength of our research is the longitudinal nature of the evaluative data, collected at the end of each ISM, providing ‘real-time’ feedback over a 6-year period. To the best of our knowledge, this is the only research that describes the development and evaluation of a training initiative delivered in the UK, focused solely on implementation science. Papers like ours are essential in order to gain a field-wide perspective on the nature and range of initiatives available so that training gaps can be identified and addressed in future capacity-building endeavours [2, 18–20, 59, 60]. A limitation of our research is that only two thirds of delegates completed our evaluation survey. However, while lower than we would have liked, this response rate was consistently achieved across the whole evaluation period enabling comparability of findings across the years.

Developing and delivering the ISM has not been without its challenges. We are still grappling with some of these issues but feel it is important to reflect on our learnings to date. Many obstacles we have encountered are interrelated; while not insurmountable, they certainly compound the complexities with building capacity in the field. We reflect on these here so they can be borne in mind by educators who may be looking to establish similar training initiatives.

A strong underlying aim of our ISM is its transdisciplinary and cross-professional approach. While this component is critical to the success of the course, such diversity can create considerable differences in perspectives and training expectations amongst delegates. While this is neither unpreventable nor unwanted (given the importance of addressing complex healthcare problems from a variety of angles), it can also be obstructive to meeting individual needs. Given our wide target audience, we could not tailor our curriculum to specific disciplines or contexts. At times, this made it hard for some delegates (particularly those with less experience) to assimilate the taught concepts and methodologies in a way that made sense to them and was applicable to their own practice setting.

Equally, an intentional feature of our ISM was to target both junior and established investigators as well as those newer to the field as many of the current training opportunities are aimed at those with more experience [26–28, 38]. This resulted in some delegates expressing a desire for sessions to be split based on levels of expertise: a view, more prevalent in the recent ISMs, as the course has grown. In 2019, we made efforts to address this through the inclusion of an ‘advanced stream’. The need to account for differential competencies for the beginner and advanced learner in implementation science has also been raised more widely in the literature [33, 61, 62], but we found it difficult deciding the level of content and specific points of focus for each stream. We also did not find any similar attempts reported in the published literature that we could use as a benchmark.

An additional aspect of our ISM was its dual focus on both implementers and researchers, which is important for two key reasons. First, training efforts in implementation science are typically aimed at researchers not implementers [32]. Second, bringing together researchers and implementers enables an open forum to raise key obstacles when implementing evidence into practice and generates research and practice discussions on how these can be overcome [19, 51]. When the ISM was initially developed in 2013/2014, core competencies for the implementation researcher and practitioner did not exist. This resulted in considerable debate amongst faculty over what topics we should include, and how. While more recently, important steps have been taken to establish curricula expectations [25, 41, 57, 59, 63] and a working draft of competencies for the implementation specialist has been produced [57], a consistent curriculum, focused on inter-disciplinary competencies, is yet to emerge [26, 27, 41, 64].

A final, but equally important challenge we have encountered is keeping up with training demands, reflected by the increasing number of delegates wanting to register on our ISM, including those from overseas. A key advantage of our ISM is that it provides efficiency in scale, attracting a range of international faculty and delegates at a singular event. In the early years, when the ISM was smaller, delegates were able to benefit greatly from the expertise of the faculty by signing up for one-on-one time to gain feedback on their individual projects. We were also able to better align individuals in the group sessions and workshops in terms of interests and experience, providing greater opportunities to discuss techniques and methods that could be applied to their specific needs. Now the ISM is substantially bigger, it has become harder to tailor it in this way, and in the interest of fairness, we no longer hold one-one sessions with faculty because we cannot do this for all attendees. This presents us with somewhat of a quandary. While we do not want to hinder the growth of the ISM, we do not have capacity to keep up with this growth if it continues to mature at its current pace. This issue resonates with wider literature that has shown there is a shortage of spaces on implementation science-focused training opportunities, with demand notably superseding availability [19, 26, 27, 38, 62]. Finding ways to build capacity in the field to reach out to a wider critical mass is essential if we are to cope with this growing demand. The online avenue holds promise, with several organisations paving the way and releasing web-based courses in dissemination and implementation science in recent years [52, 65, 66].

Finally, it is also important to note that as part of this evaluation, we did not assess whether training resulted in improved implementation of evidence-based interventions. Recent evidence has shown that training initiatives in implementation and dissemination science can lead to sustained improvements in applying evidence into practice [54], and also result in peer-reviewed publications, grant applications, and subsequent funding [26, 27, 38, 67] with scholarly productivity increasing the longer the duration of training [67]. While it was not the intention of our research to examine this, it nonetheless remains an important area of exploration to help highlight and strengthen the value and impact of training in the field. We are mindful of this and are exploring ways to assess such benefits in our future capacity-building endeavours.

## Conclusions

As evident in this article, interest in the UK ISM is growing year on year and on an international level. The development of the ISM curriculum will continue to be an annual iterative process, reflective of the evidence base as it evolves and the directly expressed needs of the delegates that attend the course. As an emerging field of interest, implementation science measures and methods are still developing [68, 69], but as new research unfolds, we will move more towards a clearer and more established consensus on teaching priorities and approaches. There is no easy formula to address some of the challenges we have faced when developing and delivering the ISM, but our consistently positive findings across a 6-year evaluation period indicate that we are at least some of the way to getting the ingredients right.

## Data Availability

The datasets are available from the authors on reasonable request.

## Abbreviations

ISM: Implementation Science Masterclass
LMICs: Low- and middle-income countries
NIHR: National Institute of Health Research
ARC: Applied Research Collaboration
CLAHRC: Collaboration for Leadership in Applied Health Research and Care
KCL: King’s College London
